# Influenza Vaccination Programs for Healthcare Personnel: Organizational Issues and Beyond

**DOI:** 10.3390/ijerph182111122

**Published:** 2021-10-22

**Authors:** Helena C. Maltezou, Eleni Ioannidou, Koen De Schrijver, Guido François, Antoon De Schryver

**Affiliations:** 1 Directorate of Research, Studies, and Documentation, National Public Health Organization, 15123 Athens, Greece; 2 Department of Internal Medicine, General Hospital of Rethymno, 74100 Rethymno, Greece; eleioannidou@yahoo.gr; 3 Department of Family Medicine and Population Health, University of Antwerp, 2610 Antwerp, Belgium; koen.deschrijver@uantwerpen.be (K.D.S.); guido_francois@skynet.be (G.F.); antoon.deschryver@uantwerpen.be (A.D.S.)

**Keywords:** vaccination, immunization, influenza, healthcare personnel, vaccination program vaccination campaign, mandatory vaccination

## Abstract

Healthcare personnel (HCP) are a high priority group for influenza vaccination aiming to protect them but also to protect vulnerable patients and healthcare services from healthcare-associated influenza and HCP absenteeism. Multi-component influenza vaccination programs targeting behavioral, organizational, and administrative barriers are critical, if influenza vaccination rates among HCP are to be raised on a sustained basis. Mandatory influenza vaccination policy is the only single intervention that can achieve high and sustainable vaccination rates in HCP in short term. In this article, we provide an overview of issues pertaining to influenza vaccination of HCP, with an emphasis on organizational issues of influenza vaccination programs.

## 1. Introduction

Influenza is a significant cause of morbidity, fatalities, and use of healthcare services globally. A recent study compiling influenza-associated excess respiratory mortality estimates from 31 countries representing five World Health Organization (WHO) regions from 1999–2015, estimated that every year an average of 389,000 (range: 294,000–518,000) respiratory deaths globally are associated with influenza [1]. Although infection control capacity has largely improved in healthcare facilities the past decade, healthcare-associated influenza and outbreaks are common, even among high-risk patients [2,3,4]. Recent studies indicate that healthcare-associated influenza is a major component of the burden of influenza in healthcare facilities, accounting for up to 17.3% of influenza cases with considerable range from season to season [5,6]. Healthcare-associated influenza is associated with complications and significant mortality mainly among vulnerable patients with comorbidities [7,8,9]. Moreover, healthcare personnel (HCP) have long been recognized as an occupational group highly affected by influenza, but also for transmission of influenza virus to their patients and colleagues and onset of outbreaks with significant morbidity, mortality, and disruption of healthcare services [3,4,10,11,12,13,14,15]. According to WHO and other public health authorities globally, HCP constitute a priority group for seasonal influenza vaccination [9,16,17,18]. The rationale for HCP vaccination against influenza relies on the need to protect them but equally important vulnerable patients, and mainly those who cannot become vaccinated because of their age (e.g., young infants) and those who cannot elicit good immunity after vaccination (e.g., immunocompromised patients, elderly) [10,19]. Influenza vaccination of HCP is also a key measure of protection of healthcare facilities from HCP absenteeism, presenteeism, and outbreaks [10,20]. Finally, influenza vaccination of HCP is an integral part of preparedness and response plans for pandemics [21]. Nevertheless, influenza vaccine uptake rates among HCP remain low in almost all countries, with the notable exemption of the United States (US) where mandatory vaccination policies were successfully implemented the past decade [10,22,23]. In this perspective we provide our view on issues pertaining to influenza vaccination of HCP, with an emphasis on organizational issues of influenza vaccination programs.

## 2. Knowledge and Attitudes of HCP towards Influenza and Influenza Vaccination

Although influenza vaccination of HCP is recommended by public health authorities for several decades, influenza vaccination acceptance and advocacy rates among HCP vary across countries and HCP professions [24]. HCP who shares strong positive attitudes towards vaccination advocacy, are more likely to recommend influenza vaccination to their patients [24]. In addition, compliance of HCP with recommendations for influenza vaccination is associated with reduced probability to decline other occupational vaccinations [25].

Recent articles indicate that there are still gaps in HCP knowledge about healthcare-associated influenza and the expected benefits of HCP influenza vaccination for them and their patients. For example, working despite being ill is common among HCP, including those working in high-risk settings. In an oncology unit, two out of three HCP with influenza continued to work despite their symptoms; approximately half of them did so because they considered their illness too minor to pose risk [4]. Similarly, a survey in a US medical center revealed that 92% of HCP with influenza-like illness (ILI) during the peak of influenza season continued to work, including HCP who provided healthcare to transplant patients [26]. These findings also indicate the need for enhancement of surveillance for prompt detection of ill employees. However, virus shedding occurs even without symptoms. A prospective, hospital-based, whole-genome sequencing study showed that 15.5% of influenza-positive swabs from HCP were collected from asymptomatic HCP [27]. HCP are often implicated in transmission of influenza virus in outbreaks in healthcare facilities [4,11,13]. Because of their comorbidities and older age, patients with healthcare-associated influenza are often at increased risk for complications and less favorable outcome [6]. Furthermore, persons with comorbidities and the elderly demonstrate insufficient immune responses after influenza vaccination [19] and therefore influenza vaccination of HCP is critical for their protection during hospitalization.

Finally, influenza is a significant cause of absence from work among HCP while influenza vaccination has a significant protective impact on it. A Canadian cohort study of 10,079 HCP of whom 77% were vaccinated, found that unvaccinated employees in winter had a two-fold increased absenteeism due to all-cause illness than vaccinated employees [28]. Imai et al. conducted a meta-analysis retrieving results from 13 published studies [29]. This meta-analysis found that compared with unvaccinated HCP, vaccinated HCP had a pooled relative risk (RR) of 0.40 [95% confidence interval (CI): 0.23–0.69] for the development of laboratory-confirmed influenza, a pooled RR of 0.62 (95%; CI: 0.45–0.85) for ILI-associated absenteeism, and a pooled shorter sick leave from work of 0.46 (95% CI: 0.71–0.21) [29]. They also reported that all published economic evaluations consistently found that influenza vaccination of HCP is cost saving based on crude estimates of avoided absenteeism by vaccination [29]. However, more studies are needed to comprehensively evaluate cost-effectiveness. Finally, a recent study of two cohorts of 37,377 patients with SARS-CoV-2 infection found that influenza-vaccinated patients had significantly less often sepsis, stroke, deep vein thrombosis, emergency department and intensive care unit admissions which indicate a potential protective effect against SARS-CoV-2 [30]. Further investigation is needed.

Gaps in knowledge and misconceptions of HCP about healthcare-associated influenza and influenza vaccine are main barriers for accepting influenza vaccination and complying with recommendations for vaccination [31,32,33,34,35]. According to recent studies, vaccine hesitancy among HCP is often associated with low-risk perception, denial of the social benefit of influenza vaccination, low social pressure, lack of perceived behavioral control, negative attitude toward vaccines, not having been previously vaccinated against influenza, not having previously had influenza, knowledge gaps about influenza, issues of accessibility to vaccination services, and socio-demographic characteristics [36]. In particular, concerns regarding vaccine safety constitute a major reason for vaccination refusal among HCP, while willingness for self-protection and protection of their family are main determinants of influenza vaccine acceptance [36].

## 3. Implementation of an Influenza Vaccination Program

Ideally, an influenza vaccination program is integrated in broader infection prevention and vaccination policies for HCP which in turn will enable a more comprehensive approach to the control of influenza, facilitate HCP compliance with vaccination recommendations, and promote a safety culture for employees and patients [37]. The implementation of an influenza vaccination program requires evidence-based knowledge, prompt design, multi-level interventions, human resources, and commitment. The influenza vaccination program should be widely available to HCP through the official website of the healthcare facility.

An annual influenza vaccination program consists of the following:

- Educational activities and communication;
- Vaccination coverage target;
- Organization of vaccination campaign;
- Analysis of vaccination data and feedback of results to HCP;
- Target achievement award.

### 3.1. Educational Activities and Communication

Evidence-based education and promotional communication are key components of successful influenza vaccination programs and are useful in modifying vaccination behavior and improving vaccination rates among HCP [38,39,40,41]. Education should be provided by well-prepared HCP teams with members from various employee categories and target all employee categories, focusing on HCP working in high-risk healthcare settings and on HCP groups with lower vaccination rates. Educational material should be evidence-based, comprehensible and adapted to the needs and concerns of employees to which it is addressed. It should also be attractive, easily accessible and revised accordingly in order to raise attention every year. Communication means can include leaflets, posters, and messages in informative systems and employee mobile phones.

Education should be provided as early as possible and cover all aspects of healthcare-associated influenza, focusing on vulnerable patients, the role of HCP, and the protection conferred through vaccination. Education about infection control measures, respiratory hygiene, and appropriate management of HCP with ILI is also imperative [42]. Information about vaccination procedures (“where and when can I get the vaccine?”) should also be communicated early in the vaccination period.

The incorporation of psychological and cognitive models ensures a holistic approach of vaccination education programs [43]. A cluster-randomized controlled trial found that the use of the preferred cognitive styles and decision-making model in internal medicine residents at Mayo Clinic was associated with significant improvement in their confidence in communicating with patients about vaccines [41]. Healthcare students also offer an excellent opportunity for early education on the benefits of influenza vaccination and for addressing potential concerns about vaccine efficacy and safety [44].

### 3.2. Influenza Vaccination Coverage Target

Setting vaccination coverage targets is a basic step for the implementation of every intervention to raise influenza vaccination rates among HCP. Vaccination coverage targets have to be feasible considering the past influenza season achievements and available human and other resources. It is suggested that an overall HCP vaccination coverage target is selected as well as specific targets for HCP per high-risk setting (e.g., intensive care units, neonatal units, oncology clinics). Influenza vaccination coverage targets should be disseminated to employees early in the vaccination period and linked to awards.

### 3.3. Organization of Vaccination Campaign

The vaccination campaign starts early before the vaccination period and peaks during the vaccination period (ideally October–November). Evidence-based knowledge and actions to surpass or eliminate barriers of HCP to comply with influenza vaccination recommendations at the level of the specific healthcare facility are critical. It is equally important to early recognize and lift barriers related to accessibility to vaccination services for HCP [38]. Beyond education, the following elements have been used in intervention programs that increased influenza vaccine uptake among HCP: free and on-site vaccination, use of vaccination reminders and/or incentives, assignment of personnel dedicated to the program, long-term plan, requiring active declination and mandatory immunization policies [38]. Mobile vaccination carts have been also successfully used [45]. Combined strategies are more effective than isolated approaches [36,38].

Active participation of the manager of the healthcare facility, designation of a coordinator and a dedicated team, assignment of concrete responsibilities to team members are critical elements of a successful vaccination program. Of equal importance is to ensure the required human and material resources for the implementation of the vaccination program. Communication algorithms should be established in advance and include all involved units and infrastructures (administrative, infection control team, pharmacy, clinics, informative systems) and involved personnel.

### 3.4. Record and Analysis of Vaccination Data and Feedback of Results

A mechanism to record vaccinations and estimate influenza vaccination coverage rates should be in place [46]. The number of employees per category and healthcare setting will allow interventions in clinics and professional categories with low vaccination rates. A mechanism to identify unvaccinated employees and send reminders and/or arrange meetings with the infection control committee is also imperative. The overall vaccination rate among HCP and vaccination rates per clinic should be communicated on a regular basis throughout the vaccination period until the end of influenza season.

### 3.5. Target Achievement Award

At the end of the influenza season, the achievement of high vaccination rates in specific clinics and units should be widely disseminated and an award should be offered to the department with the highest vaccination rate.

## 4. The Role of the Administration of the Healthcare Facility

Commitment of managers and administration to the promotion of the principle of “zero-tolerance” to healthcare-associated influenza and common values towards vaccinations is critical. The managers should also be actively involved in all steps of the influenza vaccination program, if influenza vaccination rates among HCP are to be raised on a sustained basis [38]. Meetings to discuss with departments’ directors about vaccination strategies, possible vaccination barriers, issues of maximalization of collaboration and coordination, and vaccination coverage targets are crucial. Directors should also be sensitized about their leadership role in promoting vaccination of HCP. The administration should also ensure the availability of human, education and communication resources, and appropriate technical procedures.

## 5. Voluntary or Mandatory Influenza Vaccinations: A Two Decades-Long Debate

Most countries globally rely on recommendations to vaccinate HCP against influenza [17,47]. However, after decades of implementation of voluntary vaccination policies in several countries, vaccination coverage rates rarely exceed 60.5% in almost all countries [10,31,32,33,34]. A survey conducted by WHO in 2014–2015 reported a median of 29.5% influenza vaccine uptake among HCP in 26 European Region countries [22]. In this survey, most countries had vaccination rates below 40% while in some countries decreasing trends were recorded [22].

Mandatory influenza vaccination as a condition for employment was implemented for the first time in Virginia Mason Medical Center in Seattle, US [48]. Although resistance mainly by organized employee organizations emerged, vaccination rates among more than 5000 employees rose to >98% within four influenza seasons [48]. Over the following years, many US healthcare facilities moved to mandatory influenza vaccination policies and excellent results were recorded. Concomitantly, concerns and mistrust of HCP toward influenza vaccine and administrative barriers were also addressed by facilitating on-site and free access to vaccination [48]. Influenza vaccination rates among HCP were considered as an index of safety in healthcare facilities [48]. Support from many scientific societies and leadership support were also crucial in this nationwide endeavor. In 2017/2018 for example, influenza vaccination rate was 94.8% in US healthcare facilities with mandatory vaccination policies compared with 47.6% in healthcare facilities where mandatory vaccination policies were not implemented and vaccination was not promoted or offered on-site [23]. Decade-long experience indicates that mandatory vaccination policy is the only single intervention that can achieve and sustain >90% influenza vaccination rates among HCP [37]. Studies showed the real-life benefits of influenza vaccine mandates for patients. A US study showed a significant reduction of the proportion of healthcare-associated influenza infections among immunocompromised patients in a large tertiary-care cancer center when vaccination coverage rates among HCP increased from 56% to 94% during eight influenza seasons [49]. A multi-center study conducted by Frederick et al. over three influenza seasons showed that the implementation of mandatory influenza vaccination policies was associated with a reduction by one third of symptomatic absenteeism among HCP during influenza season [20]. In particular, they found that among HCP who reported at least one sick day, vaccinated HCP had less symptomatic days of absence compared to unvaccinated HCP, and the respective OR ranged from 0.81 to 0.82 during the study period [20]. Finally, a recent population-based study found that the implementation of state laws requiring hospitals to offer influenza vaccination to HCP was associated with a 2.5% reduction in the monthly pneumonia and influenza mortality rate in the US from 1995 to 2017 (*p*-value = 0.022) during the years when vaccine strains and circulating strains matched well [50]. The largest impact was recorded among elderly and during peak influenza months [50].

European countries rely almost exclusively on recommendations to vaccinate HCP against influenza [17]. Evidence indicates that voluntary influenza vaccinations very rarely exceed uptake rates of 60–70% [10,22,37]. For example, following the implementation of multi-faced vaccination programs in England the past years, vaccination coverage rates in 2017/2018 reached 69.5% among HCP in acute and community healthcare settings, however rates ranged from 50% to as high as 92.3% [51]. Beyond implementing the right interventions and “best practices”, high adherence from staff was also recorded when influenza vaccination was embedded in an organizational culture strategy, rather than being an isolated seasonal program [51]. Significant progress has been also recorded in Ireland: influenza vaccination rates of HCP in hospitals rose from 18.1% to 58.9% over nine influenza seasons [52]. The past years very few European countries have adopted mandatory influenza vaccination policies for HCP [17,53]. Governments have a key role not only in providing political commitment on vaccinations, but equally important, in providing the appropriate legal frame for the implementation of mandatory vaccination policies.

Mandatory vaccination implies serious ethical hurdles must be taken. A person’s individual freedom must be weighed against collective goals such as obtaining herd immunity and protecting the most vulnerable patients and fellow citizens. There are many duties for HCP, both at the individual and the collective level: a duty to protect oneself, a duty to patients, to one’s family, to colleagues, and to society as a whole. Important principles here are altruism and solidarity. There are very few counterarguments and the risk of side-effects is very low. At the positive side, there is much to be gained [54]. Given the decades-long failure of voluntary vaccination programs to achieve and sustain high vaccination rates, it is imperative that mandatory influenza vaccination policies are considered ideally as a condition for employment for HCP, along with the mandatory use of masks, especially in the context of the ongoing coronavirus disease 2019 pandemic [55]. Attitudes of HCP towards vaccinations and vaccine mandates are also important and should be considered as well [56].

## 6. Conclusions

The principle of “zero tolerance” to healthcare-associated influenza is a key element of modern infection control plans. HCP are a high priority group for influenza vaccination to confer them immunity but also to protect vulnerable patients from healthcare-associated influenza and essential healthcare services from HCP absenteeism, presenteeism, and outbreaks. Multi-component influenza vaccination programs targeting behavioral, vaccine accessibility, organizational, and administrative barriers are crucial, if influenza vaccination rates among HCP are to be raised sustainably. Voluntary influenza vaccination policies have failed to achieve and sustain high vaccination rates in HCP. A mandatory vaccination policy is most probably the only single intervention that can achieve and sustain high (>90%) influenza vaccination rates among HCP.

## Data Availability

Not applicable.
